# Novel Airway Training Tool that Simulates Vomiting: Suction-Assisted Laryngoscopy Assisted Decontamination (SALAD) System

**DOI:** 10.5811/westjem.2016.9.30891

**Published:** 2016-11-08

**Authors:** James DuCanto, Karen D. Serrano, Ryan J. Thompson

**Affiliations:** * Aurora St. Luke’s Medical Center, Department of Anesthesiology, Milwaukee, Wisconsin; † University of North Carolina, Department of Emergency Medicine, Chapel Hill, North Carolina; ‡ University of Wisconsin, Department of Emergency Medicine, Madison, Wisconsin

## Abstract

**Introduction:**

We present a novel airway simulation tool that recreates the dynamic challenges associated with emergency airways. The Suction-Assisted Laryngoscopy Assisted Decontamination (SALAD) simulation system trains providers to use suction to manage emesis and bleeding complicating intubation.

**Methods:**

We modified a standard difficult-airway mannequin head (Nasco, Ft. Atkinson, WI) with hardware-store equipment to enable simulation of vomiting or hemorrhage during intubation. A pre- and post-survey was used to assess the effectiveness of the SALAD simulator. We used a 1–5 Likert scale to assess confidence in managing the airway of a vomiting patient and comfort with suction techniques before and after the training exercise.

**Results:**

Forty learners participated in the simulation, including emergency physicians, anesthesiologists, paramedics, respiratory therapists, and registered nurses. The average Likert score of confidence in managing the airway of a vomiting or hemorrhaging patient pre-session was 3.10±0.49, and post-session 4.13±0.22. The average score of self-perceived skill with suction techniques in the airway scenario pre-session was 3.30±0.43, and post-session 4.03±0.26. The average score for usefulness of the session was 4.68±0.15, and the score for realism of the simulator was 4.65±0.17.

**Conclusion:**

A training session with the SALAD simulator improved trainee’s confidence in managing the airway of a vomiting or hemorrhaging patient. The SALAD simulation system recreates the dynamic challenges associated with emergency airways and holds promise as an airway training tool.

## INTRODUCTION

Emergency airway management is a critical skill in emergency medicine. Traditional training in airway management relies on use of airway mannequins and intubations in the controlled setting of the operating room in fasting, preoxygenated patients.^1^,^2^ Neither of these methods duplicates the dynamic, challenging conditions surrounding emergency airways, including actively vomiting patients and those with blood and secretions contaminating the glottic view.^3^ Blood and vomitus in the airway has been identified as a predictor of difficult intubation.^4^,^5^,^6^,^7^ A training model that could simulate the challenges of an actively vomiting patient or a bloody airway would be ideal to prepare trainees to face these situations in real clinical practice. Here we present a novel airway training tool that simulates the airway of a vomiting patient.

The Suction-Assisted Laryngoscopy Assisted Decontamination (SALAD) simulation system pushes the boundaries of traditional mannequin-based simulations to present the trainee with the experience of using suction to control emesis and/or bloody secretions during an airway management scenario. An airway mannequin is adapted using simple hardware-store equipment to allow pumping of simulated vomit (simulated airway contaminant, or SAC) into the airway. Trainees are presented with two airway scenarios, one in which they must clear a static pool of vomit contaminating the glottic view, and one in which they must contend with continuous flow rates of SAC to suction the glottis and pass an endotracheal tube. This model has been pioneered among various trainee groups, including physicians, medical students, paramedics, nurses, and respiratory therapists. The objective of this study was to pilot an innovative airway management simulator and demonstrate learner satisfaction and self-reported comfort with difficult airways.

## METHODS

Institutional review board exemption was sought and granted. The Suction-Assisted Laryngoscopy Assisted Airway Decontamination (SALAD) simulation mannequin was built from commercially available materials. We modified a standard difficult airway mannequin head (Nasco, Ft. Atkinson, WI) to enable simulation of vomiting or hemorrhage during intubation. The modifications involved fitting clear vinyl 5/8 inch I.D. × 7/8 inch O.D. (1/8 inch wall) tubing to the existing esophagus of the mannequin, and using clear acrylic glue to secure this tubing. Quick connect hose parts were used to link the esophagus to a self-priming drill-powered fluid pump, which was connected via vinyl tubing to a large plastic reservoir that contained the SAC. The flow of SAC is controlled using a variable rheostat, which the drill is plugged into. A simple on/off switch mechanism with wireless radio control permits the instructor to control the timing and flow of SAC that the trainee must clear from the oropharynx.

We created the SAC by mixing white vinegar with xanthan gum powder, in a ratio of 10 ml of xanthan gum powder to 1L of white vinegar. Food coloring, either red or green, is added to the mixture to simulate either vomit or hematemesis. If a different consistency of vomit is desired, more xanthan gum powder could be added for thicker vomit, and less for thinner vomit. For the purposes of the study, we kept the mixture consistent. Vinegar is used to add an olfactory component to the vomit and also to help prevent the growth of mold in the system. Table 1 lists components of the SALAD simulator system and approximate associated costs.

Learners were run through two scenarios – one in which they must decontaminate a static pool of vomit in the airway prior to intubation, and one in which there is continuous vomiting that must be actively suctioned during the intubation. Students used a video-assisted laryngoscopy device (C-MAC, Karl Storz, Tuttlingen, Germany) during both intubations. The C-MAC was chosen because it allows the instructor to view the oropharynx on the video screen and provide feedback to the learner.

We used a pre-and-post session survey to collect information on learner perceptions of confidence in managing the airway in a vomiting or hemorrhaging patient on a 1–5 Likert scale, with 1 being “not at all” and 5 being “extremely.” Self-perception of skill in using suction devices and techniques during the management of emergent airways was assessed on a similar 1–5 Likert scale. We also collected learner prior experience using simulation to learn airway management. and their prior experience using simulation to learn airway management in a vomiting or hemorrhaging patient. Learner perception of realism of the simulator and usefulness of the session was also assessed using a 1–5 Likert scale after the session.

## RESULTS

Forty learners participated in the simulation, including six paramedics, five respiratory therapists, six registered nurses, seven certified registered nurse anesthetists, one nurse practitioner, six emergency physicians, seven anesthesiologists, and two medical students. Thirty-four (85%) had used simulation in the past to learn airway management skills, but only one (2.5%) had used simulation to learn airway management in a vomiting or hemorrhaging patient.

The average Likert score of confidence in managing the airway of a vomiting or hemorrhaging patient pre-session was 3.10±0.49, and the post-session score was 4.13±0.22. The average score pre-session of self-perceived skill with suction devices and techniques in the emergent airway was 3.30±0.43, and the post-session score was 4.03±0.26 (Table 2).

The average score for usefulness of the session was 4.68±0.15, and the score for realism of the simulator was 4.65±0.17.

## DISCUSSION

Blood, secretions, and active vomiting have all been identified as predictors of difficult intubation.^8^,^9^,^10^ Current airway training models use traditional airway mannequins and intubations in the controlled setting of the operating room. Trainees are then expected to apply these basic airway skills in the more complicated, real-life airway emergencies involving emesis, blood, and secretions contaminating the glottic view. These true airway emergencies occur relatively infrequently in clinical practice, so even seasoned providers often do not feel comfortable in these scenarios, adding to the stress of an already very challenging situation of a critically ill patient.^11^, ^12^, ^13^ We believe the SALAD system adds value to traditional airway teaching models by providing learners with unlimited opportunity to master the most challenging of airway skills.

While our study did not evaluate retention of skill or real-world clinical outcomes, prior research suggests that simulation is an excellent method to teach procedural competence. Simulation has been shown to be superior to non-simulation based methods of instruction in skill acquisition and retention,^14^ and also to generate a similar stress response in learners to real-world resuscitations,^15^ preparing learners to perform in high-stress situations. Retention rates of complex procedural skills after simulation training is also high,^13^ and simulation-based airway management training has been shown to improve clinical metrics such as first-pass success.^16^

The SALAD system teaches a complex set of tasks required to manage an airway contaminated with vomit or secretions. The trainee, upon opening the mannequin’s mouth and inserting the laryngoscope blade, will see the oropharynx filling rapidly with simulated vomit. Students must learn to grip the suction catheter, clear the airway of vomit, visualize the glottic structures, and pass the endotracheal tube. In the airway scenario with continuous vomiting, we instruct learners to position the suction catheter directly into the esophagus after clearing the glottic field to prevent additional contamination of the airway. This requires use of the non-dominant forearm to keep the suction catheter lodged in position, while the non-dominant hand holds the laryngoscope blade and the dominant hand manages the endotracheal tube. This requires manual dexterity, which can be quickly learned in the training sessions.

The SALAD simulation training system also allows monitoring of the learners’ progress. Skill acquisition can be easily measured and documented, as students master endotracheal tube placement while contending with low flow rates of simulated vomiting, and must demonstrate these same skills at higher flow rates. Residency and fellowship training programs can track the progression of their learners, and this can be correlated with airway milestone acquisition per Accreditation Council for Graduate Medical Education requirements.^17^

## LIMITATIONS

The primary limitation of this study is that the outcome measure was self-reported confidence with managing the airway of a vomiting patient. Additional research is needed to evaluate whether this subjective outcome translates to improved patient-oriented outcomes, such as time to intubation or success of first-pass intubation in a vomiting patient. The data show a very highly statistically significant increase in self-reported confidence for the airway management of a vomiting patient. However, the post-test was taken immediately after the training, and the possibility of skill decay is real. The duration of this improved confidence level is unknown. Additionally, this study is limited by a relatively small number of participants. Furthermore, the training exercise was multidisciplinary in nature, including emergency physicians as well as medical students, nurses, respiratory therapists, and nurse anesthetists. A minority of participants were emergency physicians, the providers arguably most likely to encounter the difficult airway in clinical practice.

## CONCLUSION

In summary, we feel the SALAD simulation system holds promise as an educational tool to provide experience in managing difficult airways. Participants’ self-reported confidence in managing the airway of a vomiting patient improved with the training session, and trainees shared anecdotal reports that the training session helped them in a subsequent clinical encounter. Further research is needed to evaluate whether training with the SALAD simulator improves patient-related outcomes in the management of emergency airways.

## Supplementary Information



## Figures and Tables

**Table 1:** Components and approximate associated costs of the Suction-Assisted Laryngoscopy-Assisted Decontamination (SALAD) simulation system.

SALAD component	Price
Nasco airway head	$895
Vinyl tubing	$19
Quick connect hose kit x 2	$6
Drill pump	$12
Corded electric variable speed drill	$20
Remote control switch	$15
Rheostat	$10
5 gallon reservoir	$10
Total simulator cost	$987
1 gallon white vinegar	$3
8 oz xantham gum	$10
Total SAC cost	$13
Total cost	$2000

*SAC,* simulated airway contaminant

**Table 2:** Pre-and-post survey results regarding simulation training system for difficult airways.

	Mean Likert score (1–5)
Pre course	
I am confident in my ability to manage the airway of a vomiting or hemorrhaging patient.	3.10±0.49
I am skilled with various suction devices and techniques during the emergent airway.	3.30±0.43
Post course	
I am confident in my ability to manage the airway of a vomiting or hemorrhaging patient.	4.13±0.22
I am skilled with various suction devices and techniques during the emergent airway.	4.03±0.26
I plan to apply the SALAD technique in the future with vomiting patients.	4.53±0.19
How useful was this session for you?	4.68±0.15
Was the simulator sufficiently realistic to challenge your skills?	4.65±0.17

*SALAD,* Suction-Assisted Laryngoscopy Assisted Decontamination
