# Supplementary material for: Novel Airway Training Tool that Simulates Vomiting: Suction-Assisted Laryngoscopy Assisted Decontamination (SALAD) System
Source: West J Emerg Med. 2016 Nov 8;18(1):117–20. doi: 10.5811/westjem.2016.9.30891 (PMC5226742; doi:10.5811/westjem.2016.9.30891)
Supplement: Supplementary file 1 [file wjem-18-117-s001.docx]

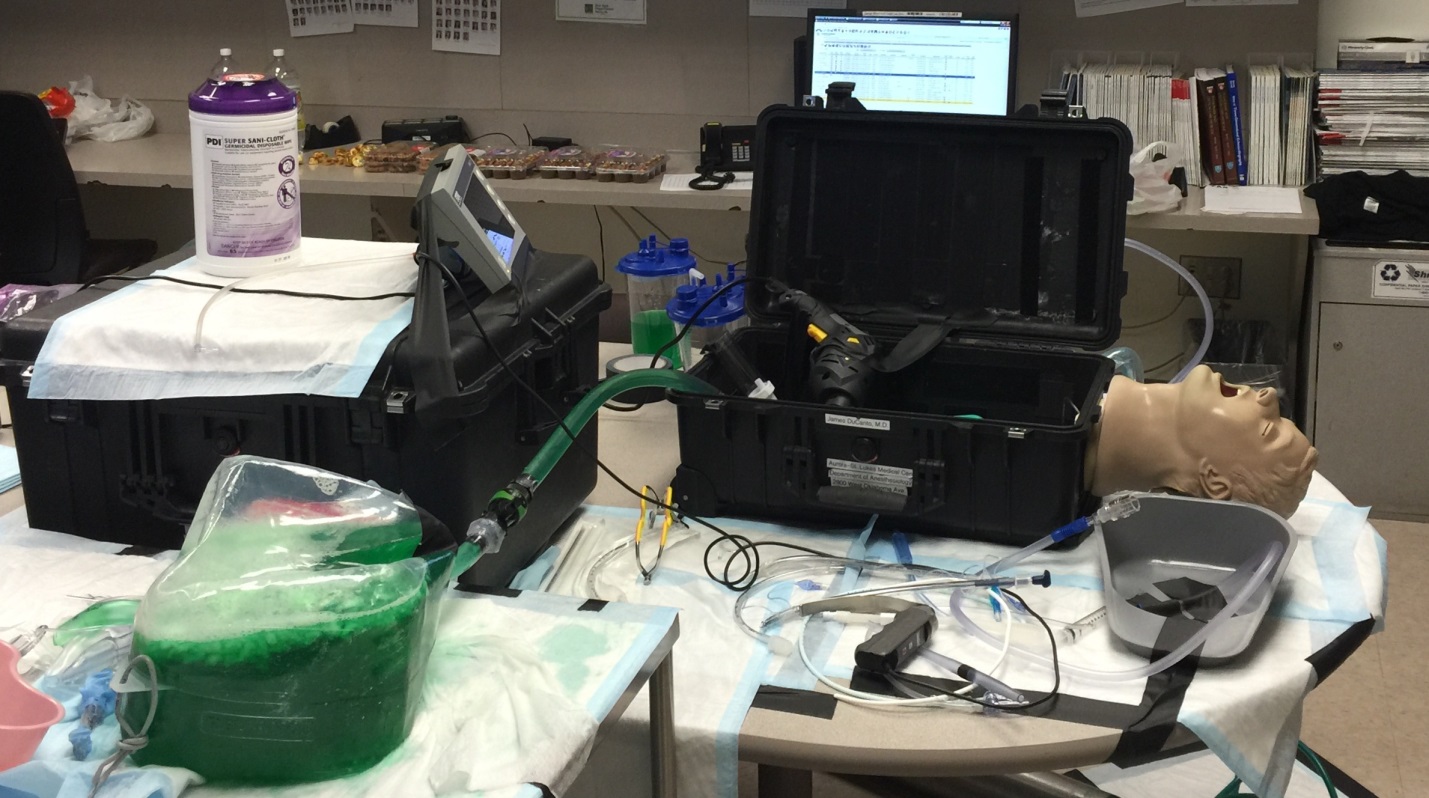
 Appendix Image 1. The SALAD simulator uses a mannequin head modified with hardware-store supplies to mimic a vomiting patient.
